# Searching for best practices of youth friendly services - a study protocol using qualitative comparative analysis in Sweden

**DOI:** 10.1186/s12913-016-1570-8

**Published:** 2016-07-29

**Authors:** Isabel Goicolea, Monica Christianson, Anna-Karin Hurtig, Bruno Marchal, Miguel San Sebastian, Maria Wiklund

**Affiliations:** 1 Unit of Epidemiology and Global Health, Department of Public Health and Clinical Medicine, Umeå University, Umeå, SE-90187 Sweden; 2 Department of Nursing, Umeå University, Umeå, Sweden; 3 Department of Public Health, Institute of Tropical Medicine, Antwerp, Belgium; 4 Department of Community Medicine and Rehabilitation, Umeå, Sweden

**Keywords:** Young people, Youth-friendly health services, Youth clinics, Qualitative comparative analysis, Primary health care, Evaluation

## Abstract

**Background:**

Swedish youth clinics constitute one of the most comprehensive and consolidated examples of a nationwide network of health care services for young people. However, studies evaluating their ‘youth-friendliness’ and the combination of factors that makes them more or less ‘youth-friendly’ have not been conducted. This protocol will scrutinise the current youth-friendliness of youth clinics in northern Sweden and identify the best combination of conditions needed in order to implement the criteria of youth-friendliness within Swedish youth clinics and elsewhere.

**Methods/design:**

In this study, we will use qualitative comparative analysis to analyse the conditions that are sufficient and/or necessary to implement Youth Friendly Health Services in 20 selected youth-clinics (cases). In order to conduct Qualitative Comparative Analysis, we will first identify the outcomes and the conditions to be assessed. The overall outcome – youth-friendliness – will be assessed together with specific outcomes for each of the five domains – accessible, acceptable, equitable, appropriate and effective. This will be done using a questionnaire to be applied to a sample of young people coming to the youth clinics. In terms of conditions, we will first identify what might be the key conditions, to ensure the youth friendliness of health care services, through literature review, interviews with professionals working at youth clinics, and with young people. The combination of conditions and outcomes will form the hypothesis to be further tested later on in the qualitative comparative analysis of the 20 cases. Once information on outcomes and conditions is gathered from each of the 20 clinics, it will be analysed using Qualitative Comparative Analysis.

**Discussion:**

The added value of this study in relation to the findings is twofold: on the one hand it will allow a thorough assessment of the youth-friendliness of northern Swedish youth clinics. On the other hand, it will extract lessons from one of the most consolidated examples of differentiated services for young people. Methodologically, this study can contribute to expanding the use of Qualitative Comparative Analysis in health systems research.

## Background

Swedish youth clinics have been responding to the health needs of young people living in Sweden for more than 40 years and constitute one of the most comprehensive and consolidated examples of a nationwide network of health care services for young people [1, 2]. However, studies evaluating their ‘youth-friendliness’ and the combination of factors that makes them more or less ‘youth-friendly’ have not been conducted.

The concept of ‘youth-friendliness’ has been developed by the World Health Organisation grounded on the realisation that, on the one hand, health care services can have a beneficial impact on youth health through providing good information for adolescents, treating those who are ill, and reaching those who are in vulnerable situations [3–5], but on the other hand, health care services are, in general, less accessible for young people and less responsive to their needs [4, 6]. The mere existence of health care services does not however ensure that young people’s health benefits from them, and, in order to achieve this beneficial effect, such services should fulfil certain criteria, namely to be accessible, acceptable, equitable, appropriate and effective for different youth subpopulations [3, 5, 7]. Health care services that fulfil those five criteria can be labelled as youth-friendly health care services (YFHS). YFHS should be grounded on nonrestrictive policies, and should be responsive to the needs of young people without prejudice and in complete confidentiality. Such services should promote youth participation and community dialogue and implement outreach activities [3, 8]. YFHS could be implemented throughout a variety of models: differentiated services for younf’g people community based actions, non-governmental organisations outreach work, health care services within schools or integrated within existing health care facilities [9–11]. One important issue when implementing YFHS is that of equality: health care services cannot be truly qualified as youth-friendly if there are certain subgroups of young people who do not feel that they are welcome and that there is an adequate response to their health needs. Another important issue when implementing YFHS is that of the sustainability of such services – in order to be effective and sustained over time YFHSs have to be integrated as part of the health system [11–14].

Making health services more friendly to young people is an important public health strategy, since despite youth being in general a healthy period, it is also a time when individuals may face greater risks of morbidity and mortality associated with violence and with reproductive and sexual health related problems. In addition, there are great inequalities in health status and in access to health care between young people living in high income countries and youth living in low income countries [3, 4, 15, 16]. In addition, youth is a period of opportunity; healthy behaviour initiated during these years will most likely remain throughout adulthood, and healthy young people have a positive transformative potential within their communities and countries [4, 17].

Despite research evidence that shows that implementing YFHS contributes to better health among young people and does not require huge financial investment, health systems worldwide have failed to implement this approach [4, 14, 17, 18]. The implementation and sustainability of YFHS face a number of obstacles such as stigmatisation, restrictive policies, untrained staff, and lack of clear health policies and implementation guidelines [11, 13, 19, 20]. Issues of equality and appropriateness are among the less adequately addressed issues [7, 9]. Even in countries where public health systems perform well, the implementation of YFHS remains a neglected area that lacks political will and clear guidelines [4, 17, 18]. Furthermore, little research has been conducted on what might be the key factors for implementing and sustaining YFHS within health systems.

### The Swedish youth clinic model

Sweden could be considered as a noteworthy exception, in the sense that the country has a comprehensive and well-established network of differentiated health care services for young people, called youth clinics (YCs). Beginning in 1970, the network of YCs was developed nationwide in order to improve young people’s access to health information and services, especially in terms of sexual and reproductive health. YCs emerged from a public concern regarding the health of young people, and were considered as a means of increasing the access of young people to health information and health care. The focus was on sexual and reproductive health issues, and this still remains as the strongest area in most of the YCs [1].

There are currently 265 youth clinics in Sweden, serving people aged 13–23, although age limits vary. Information is also provided by a web-based national youth clinic (www.umo.se) [1]. The majority of the clinics are run by county councils – some by municipalities or other organisations – and staffed with multidisciplinary teams. At the national level, most clinics are integrated in the Swedish Youth Clinic Association (FSUM). How each clinic is staffed and which services are provided varies. The minimum requirement is to have a midwife and a social worker or psychologist, with a physician working some daysof each week. The role of the midwife in youth-clinics is key, since Swedish midwives can prescribe contraceptives and they can insert intrauterine devices and implants. There are clinics that integrate physiotherapists, psychiatrists, gynaecologists, district nurses and nutritionists.

In order to improve accessibility, youth clinics are, in general located off the premises of general health services, and consultations are free of charge. In addition, there are othe promotional activities aimed at increasing young people’s awareness about the services offered by youth clinics [1]. Some clinics might offer group interventions, health education sessions, and outreach activities [2, 21]. Coordination with schools is well established and all pupils aged 13–14 visit the local youth clinics during the school year to get to know the location and the services available as well as having access to information on sex education.

The development and implementation of youth clinics has to be understood in the broader context of Swedish social and cultural norms. In general, Swedish society has a liberal attitude towards teenage sexual relations. Sexual and reproductive health issues are given priority: young people have been receiving sex education at schools since the 1950s, abortion is free on demand since 1975, and contraceptive counselling and access is easy [22]. Emergency contraceptives are sold over the counter and provided free of charge at most youth clinics; screening for chlamydia and other sexually transmitted infections is free and easy to access. Non-coercive sexual relations among young people are not negatively perceived, and policies are, in general, progressive in terms of LGBQTI (lesbian-gay-bisexual-queer-transgender-intersex) rights. However, some backlash has also been noticed: since 1990, sex education is taught less, and social segregation has increased, which might negatively impact access to information and services [23]. There is also an emerging need for primary prevention and early treatment of youth mental health problems [2, 24, 25].

Despite the existence of this network of youth clinics, young people’s health problems are a matter of concern in the country. There appear to be increasing mental health problems among young people in Sweden, as well as increases in violence, obesity, alcohol abuse, and sexually transmitted infections [25–27]. Young people from more disadvantaged backgrounds suffer more health problems, face greater risks to health and have poorer access to health services [28, 29]. Gender also plays a key role; girls and young women are at greater risk of suffering mental health problems, eating disorders and sexual abuse. Boys and young men are at greater risk of traffic injuries and suicide and have poorer access to health services compared to women. Moreover, research shows that the health problems and the help-seeking behaviour of young people are related to social aspects of gender, especially in terms of mental health and sexual and reproductive health [2, 30, 31].

A survey conducted by the Swedish Association of Youth Clinics (FSUM) showed that young people were satisfied with the care provided [32]. However, some challenges still remain, especially in terms of equity. Access to youth clinics might differ by youth subpopulation group and location. For example, boys and young men account for only 15 % of all youth clinic attendees [18, 33], migrant young people might face cultural and other barriers to access, and socioeconomic inequalities might also affect young people’s access to these services. Young people with disabilities or LGBQTI youth might face more barriers in accessing youth clinics, even if there is no published evidence on this. In addition, rural youth might find it harder to access a youth clinic since many rural municipalities lack a youth clinic.

This research proposal will scrutinise the current youth-friendliness of youth clinics in northern Sweden, and identify the ‘causal recipe’ for youth-friendliness. In addition to mapping the northern Swedish situation, this research will also help to identify the best combination of conditions – causally relevant conditions linked to an outcome [34] needed in order to implement the criteria of youth-friendliness in Swedish youth clinics and elsewhere.

## Methods/Design

This study adopts a multiple case study design, which is well suited to research of a contemporary phenomenon within its real-life context, especially when the boundaries between phenomenon and context are not clearly evident [35].

Furthermore, the case study design is appropriate for dynamic and complex situations where multiple, interacting variables may act upon intervention and outcome [36].

For the analysis, we will adopt qualitative comparative analysis (QCA), which was developed by Ragin [37]. Ragin defines comparative case studies as case studies that aim at searching for commonalities in the antecedent or causal conditions for success. In QCA, the researcher aims at finding the conditions that make the intervention work, considering them likely to be complementary or working conjuncturally (conjunctural causation). Indeed, Ragin [38] starts from the assumption that “*no single causal condition may be either necessary or sufficient for the outcome in question*” and that instead, only combinations of conditions may be found to be sufficient. In other words, there may be more than one causal combination that explains outcome (equifinal causation). As Ragin also puts it, the underlying idea is that of a “*causal recipe*”, a specific combination of causally relevant ingredients linked to an outcome [34].

For Ragin, the aim is to describe how the conditions fit together to cause an effect, or, in other words, to establish the various ways a necessary condition is met [37]. To start, an analysis is carried out on a limited number of cases that all show the outcome that is of interest [37, 39]. QCA uses Boolean algebra to assess to what extent a configuration of conditions explains outcomes in terms of *necessity* – whether the cause is present in all (or almost all) the instances of the outcome – and *sufficiency* – whether the cause is invariabily (or almost) followed by the outcome. The final step of the analysis consists in the development of a solution formula that presents the combination of conditions that best explain the outcome [38, 40–42].

In this study, we will use qualitative comparative analysis to analyse what are the conditions that are sufficient and/or necessary to implement YFHS.

We will select 20 youth clinics (cases) in order to assess the best combination of conditions to implement YFHS. First, qualitative and quantitative data will be gathered sequentially and analysed separately for each case. Second, the case results will be compared using qualitative comparative analysis.

### The setting

The 20 YCs to be included in this study constitute all the existing YCs in the four northern provinces of Sweden. This is a sufficient number for a QCA, as it will allow us to have an adequate number of cases sharing similar patterns of conditions without being too large and thus preventing us from becoming fully acquainted with every case.. We will select cases in the four northern counties for three main reasons. First, they provide a variety of youth clinics in terms of location, size and professionals at work, among other characteristics. Second, there is a degree of regional level coordination between the four counties and they share a similar geographical and cultural context, the so called Norrland region. These counties offer a third important advantage in the sense that their proximity enabled our research team to develop good relations with the key stakeholders. This is essential because the project aims at producing evidence useful for managers and professionals in charge of youth clinics by engaging them during the design and implementation phase.

The four selected counties in northern Sweden encompass 44 municipalities, 60 % of the country’s surface but only 12 % of the population; young people aged 15–24 account for 12 % of the population. Twelve of the 20 YCs are located in rural municipalities. The oldest YC was set up in 1974 in a small municipality of 18000 inhabitants, while the newest dates from 2007. On average, they have been operational for 24 years [1]. Working days and hours range from once a week in the smallest ones to five days a week in the largest ones. Age limits of the ‘target population’ differ, as well as the way mental health issues are addressed – some youth clinics focus on sexual and reproductive health, while others also offer first line mental health services for young people. All YCs are managed and funded by the county councils – who also have the responsibility for the management of primary health care centres and hospitals — while in some settings the municipality finances certain aspects and/or professionals.

### Steps

In order to conduct QCA, we will first identify the outcomes and the conditions to be assessed. The overall outcome – youth-friendliness – will be assessed together with specific outcomes for each of the five domains – accessible, acceptable, equitable, appropriate and effective through a questionnaire to be applied to a sample of young people coming to the youth clinics [5].

In terms of conditions, we will first identify what might be the key conditions to ensure the youth friendliness of health care services, through literature review, and through interviews with professionals working at youth clinics, and with young people. The combination of conditions and outcomes will form a hypothesis to be further tested later on in the qualitative comparative analysis of the 20 cases. For example, one possible hypothesis to test will be: clinics that are open more days a week, have multidisciplinary teams, motivated and well-trained staff and good referrals systems with other services will score higher on youth-friendliness. Once the information on outcomes and conditions is gathered from each of the 20 clinics, it will be analysed using QCA with fuzzy sets [34, 43].

#### Step 1: Measuring outcomes

In order to identify the outcomes, we will administer a questionnaire to assess the five domains of youth-friendliness to a sample of young people coming to the 20 YCs in the four northern provinces of Sweden. The instrument to be administered will be based on the YFHS-WHO+ questionnaire. The original instrument is based on a guide published by WHO to assess health services for young people, and on an Australian questionnaire used to assess the youth friendliness of primary care [5]. It has been validated to be used to assess youth-friendliness of first-line health care centres. It is divided into 7 subscales: access, parental support, equity, respect, privacy, absence of judgement, and quality. Since it has neither been used in the Swedish context, nor has it been used to evaluate differentiated youth health care services such as YCs, we will validate the instrument before its application.

The study universe will consist of young people (16–24 years old) users of the 20 youth clinics in the four counties in Northern Sweden: Västernorrland, Västerbotten, Jämtland-Härjedalen and Norrbotten. We will exclude people younger than 16 for ethical reasons since they are not allowed to give autonomous consent to participate. These clinics will be the primary sampling unit (PSU). Sample size was calculated using the following formula: *N* = 4 (Z_1-α/2_ + Z_1-β_)^2^ / (δ/σ)^2^ where *Z*_(1−α/2)_ = value of the normal distribution corresponding to the probability of a type 1 error of 0.05; *Z*_(1−β)_ = value of the normal distribution corresponding to a probability of a type 2 error of 0.8; δ = the difference in the mean value of the score between low and high performing clinics; σ = standard error of these means. To control for clustering by clinic, we multiplied this formula by the design effect: 1 + ρ (*m* − 1), where ρ = intra-class correlation and *m* = the number of observations per cluster [44]. A target sample size of 960 people given a type 1 error of 0.05, type 2 error at 0.8, a maximum mean difference in scores of 0.5, and intra-class correlations of up to 0.3 will be set. This would represent a sample of 48 participants/clinic. In order to take into account those who refuse to participate, the sample will be increased to 60 participants/clinic.

Data will be entered into SPSS and scores will be calculated for each of the domains as well as an overall score for each of the YCs.

#### Step 2: Identifying and assessing conditions

In order to identity potential conditions affecting the different domains of youth-friendliness, we will: 1) review the literature of YFHS as well as policy documents and reports of YCs in Sweden; 2) interview professionals working in YCs; and 3) interview young people.

The literature review will allow us to identify conditions that are potentially contributing to youth friendliness of health care services in other settings. Some important conditions that have been shown to enhance youth-friendliness are described in Table 1.

**Table 1:** WHO domains of youth-friendly health care services (modified from Tylee [4])

WHO domain	Enhancing conditions
Accessibility	• Free or affordable services
	• Convenient opening hours
	• Convenient location
	• Young people know about the services and how to get them
	• Community supports the services
	• Outreach work towards community
Acceptability	• Policies and procedures to ensure confidentiality in place
	• Attitudes of providers: provide information, support young people decision making, motivated, non-judgmental
	• Adequate environment: privacy, physical safety
	• Strategies for gathering views of young people on the services in place
Equity	• Diversity in the staff working in the service
	• Professionals treat all young people with equal respect, independently of their status.
Appropriateness	• Good referral with other services
	• Multidisciplinary teams
	• Holistic approach – looking beyond the specific reason for consultation
Effectiveness	• Professionals have the required competence
	• Protocols and guidelines exist
	• There are sufficient and appropriate equipment and resources

To our knowledge, there is no published research on this issue in Sweden, but grey literature, reports and policies from Sweden will be reviewed in order to complement the literature review.

In order to refine this preliminary list of conditions locally, we will conduct interviews with professionals working in youth clinics in northern Sweden. The COREQ guidelines will be followed when conducting and reporting the qualitative data analysis (See also Appendix). We will conduct individual interviews with around 15 professionals from different professional backgrounds, working in different YCs – in terms of i.e., size, location, and focus. In addition, and in order to gather the perspective of young people, we will conduct focus group discussions with young people who have attended YCs. Around 6 focus group discussions will be conducted. The first author and two research assistants with expertise on qualitative methods will conduct the interviews and focus groups discussions. The first author has extensive experience conducting individual interviews and focus group discussions and will closely supervise the two research assistants together with the rest of the team. Health care professionals will be invited to participate via email after a first contact with the head of the clinic. Young people will be approached through the clinic staff as well as through youth organizations (i.e., rfsu). Participants will choose the location for the individual interviews. The focus group discussions will be conducted at the university. All potential participants will be provided information about the study and the interviewer(s)/moderator before starting the interview/focus group discussion.

The final sample size of the individual interviews with professionals and focus group discussions with young people might vary and will be finally determined once saturation is reached. The interviews and focus group discussions guides will be structured in a thematic manner, inspired by the WHO domains of youth friendliness [3]. Participants will be asked to reflect on the issues that they consider important in order to ensure that strategies are put in place to deal with challenges to these domains. Participants will be asked whether they could be contacted again if needed. Interviews and focus group discussions will be audio recorded, transcribed verbatim, and analysed using thematic analysis inspired by the WHO domains of youth-friendliness [45]. Notes will be taken during and after the interview and during the analysis process. The first author and the two research assistants will be involved in the coding process and negotiate the codes between them. The software Open Code 3.4 will be used to help managing the coding process. Even if the WHO domains will be used as a starting point, we will remain open to new emerging themes. The derived themes will be further discussed with the entire research team. Preliminary findinsg will be presented to some of the participants for feedback.

From the analysis of the qualitative data from professionals and youth, a refined list of conditions that might be important to implement each of the domains of youth-friendliness will be developed. Configurations of combinations of conditions and outcomes will be developed by the team to be further tested in the next step. Afterwards, measurable indicators and instruments to collect data on the conditions selected will be created. We foresee that some of the conditions could be assessed through secondary data and reports (i.e., opening hours and number of staff), others via interview with the person in charge of the YC, and observation (i.e., physical arrangements to ensure confidentiality), while others might demand other instruments (i.e., measurement of staff motivation might demand the application of a questionnaire to the professionals working at the youth clinic). The selected conditions will be measured in each of the YCs and numeric values will be given for each of them.

#### Step 3: Identifying the best combination of conditions to implement YFHSs

Using the numeric values for the outcomes and conditions for each of the 20 cases, a table will be produced. The table will be calibrated in order to conduct a qualitative comparative analysis using fuzzy sets. Fuzzy sets are used when the causal conditions and outcomes are multichotomous namely, they vary by level of degree [46]. We will use the software programme fs/QCA to produce a raw table with calibrated values [47]. The raw table will be imported into fs/QCA to assess the combination of conditions that lead to the overall outcome of youth-friendliness, as well as to each of the specific outcomes of accessibility, acceptability, equity, appropriateness and effectiveness.

Afterwards a truth table will be produced that will allow us to assess the different combinations of conditions connected with positive outcomes. The truth table displays all of the possible combinations of conditions leading to the outcome. From the emerging truth table, the inconsistencies will be eliminated – configurations of conditions with less than one case, and the outcome will be reset to 1 if consistency is higher than 0.8. A standard analysis will be applied, and the intermediate solution formula will be obtained – based on logical reduction, but retaining conditions that theoretically contribute to an explanation [34, 48]. The solution formula will depict those combinations that are more relevant to produce the outcome. Since usually more than one combination of conditions emerges, for each of them, consistency and coverage scores will be calculated. Consistency represents the extent to which a combination of conditions leads to an outcome and ranges from 0 to 1. If a combination of conditions has a consistency of 1, this means that such a combination always leads to the outcome. Coverage represents how many cases with the outcome are represented by a particular combination of conditions. If a combination of conditions has a coverage of 1, this means that this combination is able to explain all of the occurrences of the outcome.

## Discussion

This paper describes a protocol that uses a multiple case-study design and qualitative comparative analysis to identify good practices for making health care services youth-friendly.

We foresee a number of challenges during the application of this protocol. The first challenge emerges from the way we are going to assess the outcomes, through a validated version of the YFHS-WHO+. The existing instrument has been used to assess the youth-friendliness of ordinary primary health care centres and not differentiated services, and it has never been used in the Swedish context [5]. Thus, we will need to engage in a thorough validation process to make sure that the instrument is appropriate to assess the intended outcome in the Swedish YCs’ context. Experts, stakeholders, professionals within youth clinics and young people will need to be involved [49–51]. Even if this might be time consuming we consider that besides being an imperative to make sure that the outcomes are accurately measured, it can become an opportunity to engage relevant stakeholders in the project from the start. The validated instrument can become a useful tool to be applied to youth clinics and other health care services attending to the needs of young people (school health, primary health care, youth psychiatry) in Sweden and in other parts of Europe.

The second challenge will consist in defining and measuring the conditions. QCA does not allow for the inclusion of a large number of conditions, so we will need to be careful in the selection process. The selection of conditions to be included in the configurations and tested is not a mechanical exercise, but should be grounded on theoretical and locally relevant knowledge [34, 43]. We consider that basing this selection on both existing conceptual frameworks – WHO youth friendliness domains – and locally gathered information from different sources – adds to the trustworthiness of the selection process. Measuring the conditions also poses challenges, since some of the potential conditions might not be easy to measure. A third challenge relates to the fact that youth clinics might be very homogeneous in terms of outcomes, and lack of variation will make it difficult to draw any conclusions about the best combinations of conditions.

The added value of this study in relation to the findings is twofold: on the one hand it will allow a thorough assessment of the youth-friendliness of northern Swedish youth clinics. On the other hand, it will extract lessons from one of the most consolidated examples of differentiated services for young people. The research community has failed to take advantage of the Swedish model as an interesting case study to analyse facilitators and challenges for implementing and sustaining youth-friendly services within national health systems. It is especially interesting to notice that while certain countries are trying to implement youth-friendly services following the Swedish youth clinic model (i.e., Estonia [52]), there are no published assessments of Swedish youth clinics’ youth-friendliness and the strategies used to achieve it. This study protocol aims to contribute to filling this knowledge gap by both by evaluating the situation of Swedish youth clinics against the World Health Organisation criteria, as well as extracting the key combination of conditions needed to make youth clinics youth-friendly. We claim that such ‘causal recipes’ might not only be applicable to northern Swedish clinics, but to youth clinics and other primary health care services that young people might access (i.e., primary health care centres, mental health services, school health care services) in Sweden and other similar contexts.

Methodologically, this study can contribute to expanding the use of QCA in health systems research. QCA has, as yet, not received much attention within health systems research. For health systems research, it is important to assess causality not in terms of isolated factors explaining outcomes, but in a more complex and naturalistic way where the combination of contextual conditions, intervention components and internal mechanisms combine to bring together outcomes [40]. Case-studies are useful methodologies to explore interventions and processes in a particular context. A larger numbers of cases can enrich the study by adding variation [35]. However, sometimes it becomes difficult to make sense of large amounts of information and to extract transferable results when the number of cases increases to a certain level. Qualitative comparative analysis can be useful when trying to maintain the balance between maintaining familiarity with the data as well as looking for patterns that might be transferable to other situations [34, 37, 38, 46, 53].

To come up with a ‘recipe’ for youth-friendliness is certainly appealing, especially for stakeholders. However, it can be criticised as too a simplistic way of solving a complex issue. In our defence we can claim that the process of identifying the conditions and outcomes to be entered into the potential ‘recipes’ if far from simplistic. The conditions and outcomes that will be entered into the calculations of solution formulae will be carefully chosen after a thorough process of quantitative and qualitative data collection. In addition, they will be conceptually/theoretically driven, grounded on the WHO domains. The next step, not contemplated in this protocol, should be a more in depth exploration of selected cases in order to understand how the identified ‘recipes’ work, for whom they work, and under what particular circumstances.

## Abbreviations

FSUM, Swedish Youth Clinics’ Association; LGBQTI, lesbian-gay-bisexual-queer-transgender-intersex; QCA, qualitative comparative analysis; WHO, World Health Organization; YC, youth clinic; YFHS, youth friendly healthcare services

## Appendix

**Table 2:** COREQ 32 items cheklist

Domain 1: research team and reflexivity	
1 Interviewer/facilitator	On page 10: The first author and two research assistants with expertise on qualitative methods will conduct the interviews and focus groups discussions. The first author has extensive experience conducting individual interviews and focus group discussions and will closely supervise the two research assistants together with the rest of the team. On page 10: All potential participants will be provided information about the study and the interviewer(s)/moderator before starting the interview/focus group discussion.
2 Credentials	
3 Occupation	
4 Gender	
5 Experience and training	
6 Relationship established	
7 Participant knowledge of the interviewer	
8 Interviewer characteristics	
Domain 2: study design	
9 Methodological orientation and theory	Page 11: Interviews and focus group discussions will be audio recorded, transcribed verbatim, and analysed using thematic analysis inspired by the WHO domains of youth-friendliness [45].
10 Sampling	Pages 10 and 11: In order to refine this preliminary list of conditions locally, we will conduct interviews with professionals working in youth clinics in northern Sweden. We will conduct individual interviews with around 15 professionals from different professional backgrounds, working in different YCs – in terms of i.e., size, location, and focus. In addition, and in order to gather the perspective of young people, we will conduct focus group discussions with young people who have attended YCs. Around 6 focus group discussions will be conducted. […] Health care professionals will be invited to participate via email after a first contact with the head of the clinic. Young people will be approached through the clinic staff as well as through youth organizations (i.e., rfsu). All potential participants will be provided information about the study and the interviewer(s)/moderator before starting the interview/focus group discussion. The final sample size of the individual interviews with professionals and focus group discussions with young people might vary and will be finally determined once saturation is reached.
11 Method of approach	
12 Sample size	
13 Non-participation	Will be reported once data is collected and analyzed.
14 Setting of data collection	Page 10: Participants will choose the location for the individual interviews. The focus group discussions will be conducted at the university.
15 Presence of non-participants	We expect that nobody else will be present, but in case non-participants are present, this will be reported.
16 Description of sample	Demographic data will be collected in order to describe the sample (age, gender, years working, professional background, level of studies, ethnic background).
17 Interview guide	Page 11: The interviews and focus group discussions guides will be structured in a thematic manner, inspired by the WHO domains of youth friendliness [3]. Participants will be asked to reflect on the issues that they consider important in order to ensure that strategies are put in place to deal with challenges to these domains.
18 Repeat interviews	Page 11: Participants will be asked whether they could be contacted again if needed. If repeat interviews are conducted, this will be reported.
19 Audio/visual recording	Page 11: Interviews and focus group discussions will be audio recorded,
20 Field notes	Page 11: Notes will be taken during and after the interview and during the analysis process.
21 Duration	The duration of the interviews will be stated.
22 Data saturation	Page 11: The final sample size of the individual interviews with professionals and focus group discussions with young people might vary and will be finally determined once saturation is reached.
23 Transcripts returned	We do not plan to return the transcripts to the participants unless something is unclear.
Domain 3: analysis and findings	
24 Number of data coders	Page 11: The first author and the two research assistants will be involved in the coding process and negotiate the codes between them. The software Open Code 3.4 will be used to help managing the coding process. Even if the WHO domains will be used as a starting point, we will remain open to new emerging themes. The derived themes will be further discussed with the entire research team. Preliminary findings will be presented to some of the participants for feedback.
25 Description of the coding tree	
26 Derivation of themes	
27 Software	
28 Participant checking	
29 Quotations presented	Will be reported once data is collected and analyzed.
30 Data and findings consistent	
31 Clarity of major themes	
32 Clarity of minor themes	
